# A Thin Line: Governmental Border Communication in Times of European Crises

**DOI:** 10.1111/jcms.13398

**Published:** 2022-08-15

**Authors:** Verena K. Brändle, Olga Eisele

**Affiliations:** ^1^ Department of Communication University of Vienna Vienna

**Keywords:** borders, crisis, government communication, Europe, latent semantic scaling, qualitative analysis

## Abstract

In response to the recent crises in Europe, many governments have tightened their border controls despite considerable criticism from the EU Commission and civil society. While borders are at the core of recent crises, we lack systematic evidence of how governments publicly inform about border politics and justify measures. Therefore, we ask: How do EU governments communicate about borders? We analyze a comprehensive sample of press releases of the Austrian and German governments over 12 years (2009–2020). Applying a mixed‐methods design, we employ automated text analysis, specifically latent semantic scaling (LSX) to scale documents regarding how they communicated permeability (openness and closedness) of borders and the state of affairs regarding a state of crisis and routine. Based on this quantitative analysis, we then apply qualitative text analysis to explore the nuances and patterns of this communication to gain in‐depth insights into governmental stances about borders.

## Introduction

Reinforced in the post‐World War II era, national borders are highly accepted institutions for state sovereignty internationally. For the EU, with its initial foundation during that phase, bordering coupled with integration processes has shaped the lives of millions of people, both residents within the EU and those aspiring to obtain residency or EU citizenship status.

While the early 2000s were described as a phase of stability not least through border consolidation (Simmons, 2019), the last decade highlights a renewed volatility of European borders associated with political or socio‐economic instability: Crises such as Brexit, the EU and its Member States' handling of migration since 2014, the COVID‐19 pandemic, and most recently political considerations to fast‐track Ukrainian EU membership amidst a Russian war of aggression all raise crucial questions about the role of national borders within the asynchronous process of European dis‐/integration.

The role of national governments as executives to implement border politics and especially how governments legitimize the many changes in border permeability are hereby often‐ignored issues. Despite the central role of governments for borders, we hardly have any systematic evidence on how national governments in the EU communicate about borders to the public, thereby (re‐)defining and signalling borders. We therefore do not know what information governments provide and, thus, how they justify their decisions regarding borders.

Asking broadly how EU governments communicate about borders, we explore communication that ranges from restrictive borders/bordering to instances where borders, and especially cross‐border activities, are encouraged. Given the novelty of this perspective, we rely on an interdisciplinary body of scholarship. Drawing from border studies, we understand ‘the border’ as a political construct, which can be instrumentalized for different purposes, such as filtering, and which exists in various forms, from violence to the enabling of freedom of movement, from isolation to collaboration (Johnson et al., 2011). Borders are then never entirely closed nor entirely open, but range on a scale of permeability (Paasi, 2012, p. 2307).

Specifically, we investigate how such dynamics manifest in governmental border communication. Drawing from literature on government communication as a subfield of political communication and democratic theory, we understand governmental border communication as an instrument for governments to strategically give meaning to borders that serves their specific agendas across different policy issues, and so, to legitimize their border politics in the broader public arena (see Canel and Sanders, 2012; Sanders, 2020). Governmental border communication is consequently part of providing accountability over governmental decision‐making about the boundaries of the state and, as we will see, also the boundaries of the EU.

Methodologically, we explore German and Austrian governmental press releases over the years 2009–2020, a period marked by a dynamic mix of several crises. While both EU Member States share experiences of EU crises, they also differ in respect to stability in their government formations, with slightly differing versions of Merkel governments for Germany and various governmental scandals and coalitional changes, including an ad‐hoc expert government, in Austria. Applying a mixed‐methods design, we first employ automated text analysis, specifically the novel latent semantic scaling (LSX) (Watanabe, 2021), as a semi‐supervised approach to scale press releases regarding how they communicated permeability (degrees of openness and closedness) of borders and the state of affairs (degrees of normalcy and crisis). Based on this quantitative analysis, we then analyze border communication qualitatively to gain a deeper understanding of what kinds of patterns and understandings about borders governments communicate in relation to which states of affairs.

Overall, our research provides a new perspective on bordering in the form of strategic government communication. Based on an inductive‐exploratory approach, our analysis of 12 years of border communication strongly indicates that it does not strictly unfold along the lines of defined cosmopolitan or communitarian conflicts (for example, de Wilde et al., 2019) or differentiate clearly between internal‐EU debordering and external‐EU rebordering (Schimmelfennig, 2021). Instead, for governments, it is strategically useful to do both, often at the same time, to legitimize their changing political decisions in response to more short‐term goals. Our findings therefore suggest that governments do not necessarily stick to a specific ideological position about either openness or closedness of borders in their communication. Instead, we suggest that bordering in government communication can be understood as a complex process, attempting to legitimize the often short‐term, responsive, and at times conflicting dynamics of border politics.

## I Approaches to Borders

The study of borders is characterized by a growing, interdisciplinary body of literature. From a state‐centric perspective, borders are a constitutive part of a state's prerogative for self‐determination, binding sovereignty to geographical territory, as outlined in the Treaties of Westphalia in 1648 (Osiander, 2001). The permeability of borders, most generally, relates to questions of whether, how and why states co‐operate. European integration scholars have recently returned to analyzing the dynamics of bordering processes. ‘Debordering’ (Schimmelfennig, 2021) within the EU is considered to have weakened national competencies (Scipioni, 2018), fuelling political and social cleavages as well as Eurosceptic party successes in Europe (Kriesi et al., 2006; Hooghe and Marks, 2009). Increasingly, scholars also point to ‘rebordering’ of external borders that is shaping European dis‐/integration processes today (Schimmelfennig, 2021). Finally, scholars also consider the politics of borders to lie at the core of an observed divide in Europe since the early 2000s: the ongoing contestation and polarization between liberal cosmopolitan and exclusive/nationalist, and communitarian ideologies (for example, de Wilde et al., 2019). Open borders hereby signify tolerance, diversity and a focus on international collaboration, while closed borders indicate protectionism and a focus on national identity.

State‐centred scholarship tends to understand borders as manifest demarcation lines of territory. Their permeability is often described in binary terms such as rebordering and debordering practices or communitarian and cosmopolitan politics. These categories enable research to explain processes of European dis‐/integration or ideological conflicts about border issues. In other words, in state‐centric approaches, borders are more often taken for granted and their legitimation only considered in questions of governance beyond the state.

This understanding of borders as taken‐for‐granted institutions has received criticism from critical, constructivist and poststructuralist scholarship, described under the umbrella term of ‘border studies’. This literature focuses instead on the symbolism and meaning of borders, for example, as expressions of authority, power or violence. Border studies considers the border not merely as a describable demarcation line (dissolving or not) between different states but as a process and practice that gain meaning from being performed, accepted, rejected, recognized or acted upon (Paasi, 2012, 2020). Borders are hereby understood as social and political constructs of power, not automatically markers for the boundaries of society, culture and/or identity (Agnew, 1994). They are dynamic, filtering and context‐contingent (Johnson et al., 2011, p. 65), constitute processes of ‘b/ordering and othering’ across different territorial scales (van Houtum, 2005), and construct binary categorizations such as elites and ordinary citizens, or insiders and outsiders (see Rumford, 2007).

In this article, we focus on governments (not entire states) as particularly powerful, yet under‐explored actors when it comes to border‐related decision‐making. As executive branches, they make and implement decisions about border permeability and have the competencies to execute and negotiate ad‐hoc closures and openings in specific circumstances, such as national security or reinstating freedom of movement. For these purposes, governments constantly engage in the construction of the meaning of borders, their renegotiation and their justification to the public.

## II Introducing Governmental Border Communication

Turning to the last decade of socio‐political turmoil in Europe, it is safe to say that European crisis events have played an important part in charging the dynamics of border politics. While temporary internal EU border controls for migration control were uncommon in the early 2000s (Groenendijk, 2004), they have become a typical feature in the last years. These dynamics in the last decade arguably raise doubts over border politics following clearly distinguishable rebordering and debordering processes or straightforward cosmopolitan of communitarian positions. Instead, we need to adapt a more pragmatic approach to understand government communication about borders in the last decade.

The link between crises and borders is hereby not new. Historically, territorial claims tend to cluster around times of systemic instability (Abramson and Carter, 2021). On the one hand, crises are situations of uncertainty and contingency, often requiring fast solutions. In that sense, they are often described as an ‘hour of the executive’ during which democratic checks and balances are suspended to accelerate executive decision‐making (Merkel, 2020). During EU crises, some governments fell back on unilateral decision‐making to find solutions instead of relying on often slow and incremental supranational co‐operation, potentially overriding vital national interests, while also demanding a rebordering of external EU borders. This was, for example, visible during the 2015/16 political crisis about migration to negotiate an EU‐wide scheme for distributing responsibility for taking in asylum seekers and processing their asylum applications (Kriesi et al., 2021), or the ad‐hoc decisions by several EU governments to no less than close their borders to contain the spread of the COVID‐19 virus within their countries (Genschel and Jachtenfuchs, 2021).

Governments also encourage and ‘open’ borders for cross‐border activities, such as cultural exchange, tourism, sub‐regional collaboration on infrastructural/logistical investments, and biodiversity conservation policies, since they are economically beneficial for border regions and the state (Spolaore, 2016; Basboga, 2020). For example, as we witnessed in the last years, freedom of movement within the EU is granted in times of stability but can be revoked in situations of risk. Furthermore, certain crises, like the financial crisis, for example, did not result in a redefinition of physical borders as this would not have ‘solved’ any of its emerging problems.

Against this background, we argue that to justify their border politics, governments need to communicate about borders pragmatically, and thus strategically. In other words, the ways in which governmental actors decide to handle border permeability in relation to diverse issues, such as security issues or environmental collaboration, is a matter of political and communicative strategy. Focusing on the latter in this article, government communication specifically describes communication by actors from the executive branch. This type of communication is necessary to inform and democratically engage the public, and ideally achieve acceptance of policies and decisions as well as accountability (Sanders, 2020).

We define border communication as government communication about border politics to the public in relation to the permeability of borders. As such, border communication represents the end‐product of internal strategizing and negotiation as an official position (see Schmidt, 2008, p. 308). Governmental border communication therefore provides insight into how governments give meaning to borders in the face of various political interests (for example, economic growth, technological innovation as well as national security), which require open and closed borders at the same time. More generally, border communication makes governmental understandings of borders and their discursive construction for legitimation purposes visible. Therefore, to answer our research question, we first need to clarify whether governments' border communication is significantly different during crises than in times of stability or routine politics and in which way this border communication relates to degrees of border permeability (scale between open and closed borders). After this step, it is necessary to analyze in‐depth the construction of the meaning of borders, their permeability and with which kinds of situations they are associated.

## III Methodology

Given the novelty of our research topic, we follow an inductive‐exploratory approach, relying on a mixed‐method design. We focus on two specific governments as cases and explore both broader trends over time and in terms of content. We also conduct an in‐depth analysis of the nuances of governmental border communication.

### Cases

From 2009 to 2020, EU governments have been dealing with a considerable number of crises, such as the economic recession, the migration crisis, Brexit, the climate crisis and the COVID‐19 pandemic. Our study focuses on Austria and Germany. As EU Member States, they are embedded in the same regulatory frameworks about borders (Schengen area) where ‘re‐/debordering’ of Europe has been a constant issue during the last decade of crises (Schimmelfennig, 2021) that we have described above. While many such crises have contributed to the already growing prominence of right‐wing populism across Europe (Caiani and Graziano, 2019), both countries are also great beneficiaries of freedom of movement and open border politics during stability. Tourism, intra‐EU labour migration, export of goods, and socially and culturally intertwined local border areas all might still be easier to justify but nevertheless require communicative adaptation in the face of reinvigorated right‐wing parties.

Beyond these similarities, we also find important differences regarding government formation and coalitions over the period of analysis. Austria's decade has been much more volatile in this respect, since it has undergone several changes in government within the 12 years under scrutiny. The migration crisis contributed to the resignation of the Social Democratic Austrian Chancellor, giving way to a coalition between the Austrian People's Party (ÖVP) and the right‐wing Freedom Party (FPÖ). This government was finally replaced by several expert governments in 2019 and a coalition of the ÖVP and the Green Party in 2020. In Germany, in contrast, government formation was dominated by Angela Merkel's chancellorship. After governing with the liberal Free Democratic Party (FDP) from 2009 to 2013, her Christian Democratic Union (CDU/CSU) formed a coalition with the Social Democrats (SPD). This grand coalition has lasted since 2013, ending with Merkel's decision not to stand for office in the 2021 elections. Overall, then, the two cases studied are particularly suitable regarding an exploration of border communication because of the ways in which they were affected by several crises and governmental dynamics.

### Sample and Mixed‐Methods Design

In order to analyze the border communication of the Austrian and German governments, we draw on an extensive dataset of press releases (Eisele et al., 2022). Press releases can be understood as strategic attempts by governments to influence the media agenda (Kiousis et al., 2006, p. 267). In this way, government communication presents official positions that governments wish to portray after internal negotiation, displaying how they justify and construct specific themes at hand (see Schmidt, 2008, p. 310).

We included all ministries and the chancelleries of both countries from 2009 to 2020 and scraped all press releases from their respective websites or repositories, relying on the *R* package *rvest* (Wickham, 2019a). We used regular expressions as well as the *stringr* (Wickham, 2019b) and *lubridate* packages (Grolemund and Wickham, 2011) to clean and prepare the text data for computational text analysis. To name a few, data from different repositories, for example, had different date formats which needed to be adapted to one overall format for the whole dataset. Html features, for example, indicating the original format or function (for example, headline) of a text part, needed to be removed. The number of press releases amounts to a total of *n* = 55,365 (*n* = 27,681 for AT; *n* = 27,684 for DE).

We employ a mixed‐methods approach (Burke Johnson and Onwuegbuzie, 2004), combining automated quantitative text analysis of a large dataset of 12 years to analyze overall trends with qualitative analysis to gain in‐depth insights into governmental border communication. This design enables us to balance the weaknesses and strengths of qualitative and quantitative research (Blaikie and Priest, 2019).

For the quantitative text analysis, we used latent semantic scaling (Watanabe, 2021), implemented in the LSX package in R, to understand how the topic of borders is discussed in governments' press releases (see Rauh, 2022 for a similar approach). LSX combines a dictionary approach with word embeddings. It is a self‐referential approach in that it does not rely on algorithms pre‐trained on external material. Instead, LSX learns about the semantic content of governmental communication in press releases and creates a semantic space in which the contents of press releases are located based on co‐occurrences of words in the corpus that is analyzed. The LSX algorithm is trained on sentences, and ideally, there should be around 200,000–400,000 sentences included in the training to obtain a valid representation of word vectors for the calculation of semantic proximity (Watanabe, 2021, p. 87).

To detect the topic of interest (borders) in press releases, we first used a central defining target term to create a set of model terms. Model terms are words that are significantly associated with the target term; in their entirety, the target and model terms represent the topic and are used like a dictionary to detect relevant instances in press releases. In our case, we used the target term *grenz* (border) to identify model terms in the corpus which are significantly associated, i.e., co‐occurring, with ‘border’ at *p* < 0.001. We cleaned the terms to ensure a valid representation of the border theme [that is, excluding words that contain *grenz* but do not relate to borders, such as ‘Obergrenze’ (upper limit)]. This pre‐processing step was discussed extensively among the authors to ensure reliability and validity of the model terms (see Appendix Tables A1 and A2). In a second step, the word embeddings approach implemented in LSX was used to scale the borders topic according to the (1) permeability in terms of an open or closed connotation of borders in the text; and the (2) state of affairs, probing if borders are discussed rather with reference to crises or in a normal and routine manner. This scaling is the average of semantic proximity of the sentences per text, situating it between the defined poles. How semantically close individual press releases about borders are, for example, to openness in contrast to closedness, is calculated based on the semantic space defined by word co‐occurrences as explained above.

Semantic proximity of sentences in the analyzed texts is calculated with reference to a small set of defined seed words representing each pole (normal vs. crisis; open vs. closed, see Table 1). An aggregate, normalized score of polarity per document (see Watanabe, 2021, p. 87ff.) is given, serving thus as an indicator of how semantically close to a certain pole the text is on average; for example, how close a text is to the pole ‘open’ would be indicated by positive values as sentences are on average semantically more proximate to the seed words we have defined for openness. Seed words should be generic words that can be used across projects; they can be selected using a thesaurus, but given the self‐referential nature of the model, we also checked seed words with keyword‐in‐context analysis of the border terms in our data to ensure validity (see Table 1).

**Table 1:** Seed Words Permeability (Openness/Closedness) and State of Affairs (Normal/Crisis)

	German original for analysis	English translation
openness	offen, öffnung, geöffnet, keine	open, opening, opened, no(‐ne)
closedness	schließen, geschlossen, zurückweisen, kontrolle	close, closed, reject, control
normalcy	routine, normalität, alltag, entspannt	routine, normalcy, every‐day, relaxed
crisis	krise, ernst, not, angespannt	crisis, serious, emergency, tense

Validity can also be qualitatively inspected by plotting the model terms regarding how frequently they occur in the corpus and where between the defined poles they are located on average. In this way, we can shed light on if the term ‘Außengrenze’ (external border), for example, is, on average, located closer to ‘open’ or ‘closed’ in governmental press releases.

While Austria and Germany are both German‐speaking countries, we still performed analysis separately on the Austrian and German corpus to accommodate linguistic specificities. After completion of the two steps of analysis, that is, the dictionary analysis (border theme) and the polarity scaling (open vs. closed; normal vs. crisis), we dropped such press releases from the dataset that mentioned our model terms less than 10 times, resulting in a subsample of *n* = 9,949 press releases for Austria and *n* = 7,631 press releases for Germany, representing press releases discussing borders. As the sample would have been too small to perform the LSX analysis only on press releases covering the border theme, we decided to use the whole corpus and sample border press releases *post hoc*. 1

The qualitative analysis builds on and validates this analysis by investigating in a more detailed manner how the German and Austrian governments publicly define and portray border‐related issues, and so make the discursive process by which openness and closedness of borders are constructed visible. To break down the empirical material for a qualitative analysis, we selected those 40 press releases that yielded the highest scores in the polarity scaling of the LSX for degrees of openness (*n* = 20) and for closedness (*n* = 20) for each case. We also selected such press releases that contained both the terms *grenz* (borders) and *krise* (crises) from the full sample. In this way, we reduced the risk of relying too much on a narrow understanding of ‘Grenze’ in terms of border control or migration. This sampling also enabled us to explore whether the ‘crisis’ theme emerged in relation to borders from the data. Also here, we screened for and excluded press releases that merely contained words such as ‘Grenzwert’ (limit for a value). In this way we accounted for both ‘open’ and ‘closed’ meanings of borders and, at the same time, created a sample that was narrow enough to focus on territorial borders (instead of, for example, bordering between social groups).

Based on this purposive sample, using MAXQDA, we developed an inductive coding scheme, analyzing what kinds of topics, themes or concepts emerged in relation to them (see Appendix Table A3). Our focus was on the permeability of borders, specifically coding what kinds of borders governments refer to and what kinds of themes, including crises, they communicate in relation to these borders. We followed a pragmatic approach to inductive coding, as suggested by Saldaña (2011, p. 177f.): ‘[T]o understand the diverse patterns and complex meanings of social life’, we made use of several tools, such as the occurrence of specific words, frequencies and textual representation in the form of data matrices to support the inductive process. Thus, the aim was not to strictly follow the guidelines of specific discourse analytic approaches but to be able to make the most plausible interpretations. Here we also acknowledged that different ministries might address border issues in different ways and prefer different degrees of openness/closedness. Our understanding of open or closed borders is therefore not binary or defined as a dichotomy but acknowledges that it might be gradual, also composed of contradictions even within governments.

## IV Findings

We find that both governments differ in their border communication, with a focus on security in Austria, and more emphasis on cultural exchange in Germany. We find similarities in communication regarding openness. The qualitative analysis nuances these findings, showing that especially the migration crisis brings out the Austrian government's focus on external border protection and the German government's focus on transboundary crises. Furthermore, the analysis suggests that openness is still communicated through a theme of control and a description of Europe as ‘borderless’ not necessarily fitting.

### Permeability and State of Affairs: Broader Trends in Border Communication

**Figure 1:**
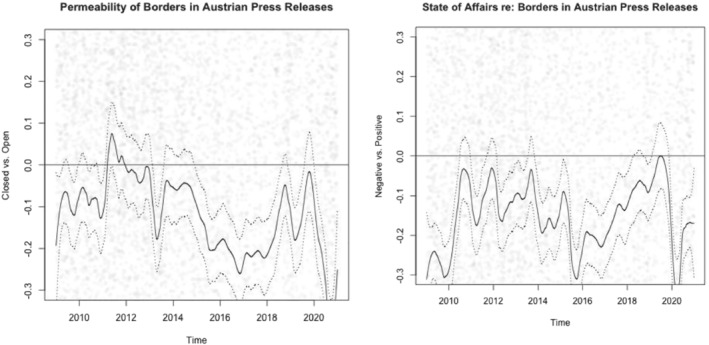
Permeability and State of Affairs in the Austrian Government's Press Releases Note: Dotted lines represent 95% confidence intervals.

Looking first into the case of Austria (see Figure 1), the LSS analysis regarding the openness of borders as discussed in press releases reveals an overall rather negative trend over the 12 years under study. It recovers in 2018, potentially influenced by the Austrian government's Presidency of the Council of the EU, but shows a steep dip again with the beginning of the pandemic in 2020. Regarding the state of affairs, a similar trend is visible; for the time around the migration crisis in 2015 and the first months of 2020, we again see a clear dip: border closures in Europe peaked in March 2020 after the WHO had declared a pandemic status and Europe to have become its epicentre (Shiraef et al., 2021; WHO, 2021). The two scores are significantly correlated (Pearson's *r* = 0.34; *p* < .001), suggesting that the Austrian government often draws a connection between the permeability of borders and crises in their press releases in our period of analysis.

**Figure 2:**
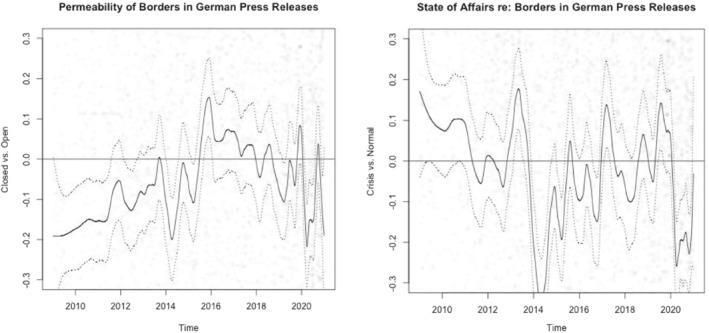
Permeability and State of Affairs in the German Government's Press Releases Note: Dotted lines represent 95% confidence intervals.

For Germany (see Figure 2), the openness connected to the topic of borders reveals somewhat different patterns, moving from statements about more ‘closed’ borders towards more ‘open’ borders, especially in the wake of the migration crisis around 2015. Also for Germany, the COVID‐19 pandemic leads to less permeability in the border discussion. The state of affairs dimension in the border discussion is relatively volatile compared to Austria, with a clear dip in 2014, slowly recovering with the migration crisis, turning downwards again with the pandemic; the two scores are significantly, but only weakly, correlated (Pearson's *r* = 0.06; *p* < 0.001). Thus, the two dimensions are discussed relatively independently in German government communication about borders, suggesting that crises do not have a great influence on governmental decisions about borders' permeability.

Regarding differences between the two countries, both dimensions of the border discussion appear slightly more balanced towards negative values in Austria than in Germany (see Table 2). In addition, we also conducted Welch two‐sample *t*‐tests to understand if Austria and Germany overall differ from each other regarding the permeability and state of affairs in which governments portray borders in their press releases. We find significant differences for both permeability (*t* = −9.4624, *df* = 16,705, *p* < 0.001) as well as state of affairs (*t* = −9.4535, *df* = 15,616, *p* < 0.001). This suggests that the two governments have different approaches in how they discuss borders regarding how permeable they are and how borders connect to crises.

**Table 2:** Summary Statistics

	Mean	SD	Max	Min	Obs
**Permeability**					
**AT**	−0.12	0.77	2.59	−5.46	9,949
**DE**	−0.01	0.74	3.48	−6.56	7,631
**State of affairs**					
**AT**	−0.15	0.74	3.26	−3.82	9,949
**DE**	−0.04	0.81	2.11	−5.76	7,631

**Figure 3:**
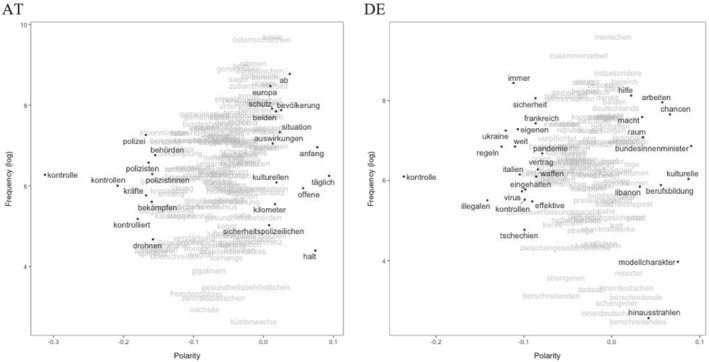
Polarity Frequency Plots for Permeability Scaling

**Figure 4:**
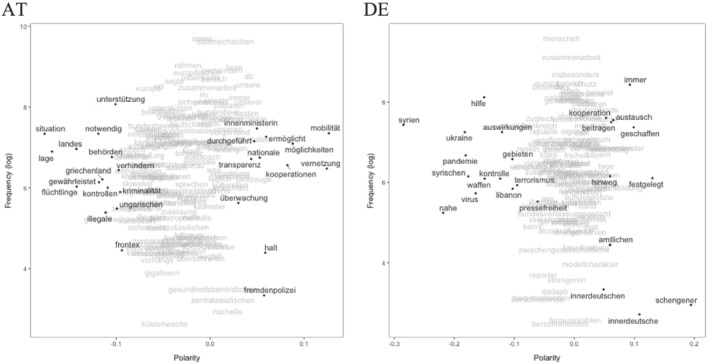
Polarity Frequency Plots for State of Affairs Scaling

Plotting the most frequent model terms (frequency) and their position in terms of proximity to our seed words (polarity) allows for a qualitative inspection of the semantic contexts of poles (see Watanabe, 2021). Thus, we look into where the terms we have used to define the border topic (model terms, see Section III) are located regarding the closed/open polarity (Figure 3) or a state of crisis vs. normalcy (Figure 4) to better understand the semantic context for different ends of these dimensions.

In both countries, the terms related to closedness of borders refer to security issues. Austria hereby focuses strongly on the implementation of control (for example, police (‘Polizei*’) and fight (‘bekämpfen’). Germany also highlights control and rules (‘Regeln’), yet also has a more international focus, referring to other crises [for example, pandemic (‘Pandemie’)] and their international dimensions in other countries (see Figure 3, polarity < 0). Openness of borders is rather discussed in terms of chances, exchange and co‐operation, potentially pointing more strongly to internal/national EU borders. Austria still focuses on the security dimension in these regards [‘sicherheitspolizeilich’ and protection (‘Schutz’)], mentioning also a European dimension. Germany focuses more strongly on an economic dimension [for example, labour (‘Arbeit’)] (see Figure 3, polarity > 0).

Plotting the model terms most closely connected to a state of crisis (see Figure 4) in Austrian press releases shows a focus on refugees (‘Flüchtlinge’), control, crime (‘Kriminalität’), and a focus on Greece with its external EU borders and related support (‘Unterstützung’). Germany, in contrast, has a much more international focus, emphasizing terrorism (‘Terrorismus’), fight, and Syria or Ukraine. On the ‘normalcy’ side of the border discussion (polarity > 0), both governments discuss development, exchange of experiences (‘Austausch’) and co‐operation (‘Kooperation’). Austria focuses on police security here as well. Overall, differences between countries are more pronounced on the crisis side as far as the state of affairs in border communication is concerned. Furthermore, Austria is generally more concerned with security issues.

Overall, the quantitative analysis of 12 years of government communication suggests that the two governments communicate about borders in a significantly different way. Delving deeper into model terms and their polarity, then, supports this conclusion, but also shows similarities, especially for the open and routine sides. Here, the terms seem to be quite similar with a focus on exchange, chances and co‐operation, thus emphasizing the advantages of open borders for specific purposes. In particular, when borders are discussed in a context of crisis (polarity of state of affairs scaling < 0), we find that Austrian and German governments emphasize different aspects: Austria is more focused on the impact of increased migration at the EU's external borders, while Germany focuses on the war in Syria more directly and less explicitly on borders.

### Nuances of Border Communication

To better understand the nuances of the similarities and especially differences in this polarity pattern that has emerged from both governments' border communication, we require an in‐depth investigation. First, providing an overview about the coded material, communication by the Federal Foreign Office dominates the German sample, followed by the Ministry for Development. The Austrian case was dominated by press releases from the Chancellor's office, the Ministry for European and International Affairs, and the Federal Ministry of the Interior. 2 We find that both the Austrian and German governments evoke an idea of ‘border’ as a political urgency during crises.

The most obvious case is the migration crisis since 2015/16. Here, our analysis illustrates how the migration crisis has provided fuel for governmental communication about border protection, suggesting how borders are, of course, an integral part of the (EU) migration regime, enabling the politicization of migration. Unsurprisingly, this differs from other crises, such as the economic crisis, climate crisis and environmental pollution, crisis as war or conflict, or the COVID‐19 pandemic. Although both governments show such patterns, the focus on border protection is particularly visible in the Austrian case. In comparison to Germany, we find a hyperfocus on border protection, especially external EU borders, border controls and asylum issues there. In particular, the Austrian government repeatedly embeds security issues at the outer‐EU border within more humanitarian‐minded statements. Sebastian Kurz, then Foreign Minister, stated 3:

Europe is required to stop the dying in the Mediterranean. We have to use the current buffer from the closure of the West Balkan route and the agreement with Turkey to find a European solution. This is what I will work towards in today's EU’ s Council of Foreign Ministers in Luxembourg. **We should no longer leave the decision about who makes it to Europe to criminal gangs of smugglers.** The rescue in the Mediterranean should also not be tied to a ticket to central Europe. **We therefore must fight illegal migration and criminal smuggler gangs and properly protect outer‐EU borders.** At the same time, we should provide a lot more support on site to open up legal flight routes via resettlement programmes and exert more pressure on origin countries. (AT, 1, 2016)

Four years after Kurz's demand to protect the EU's external borders, the current Interior Minister (in 2020) still evokes the need for ‘efficient border protection’. This demand is a constant in Austrian government communication. It remains unchanged despite an extended mandate of Frontex for border protection since 2016 and a considerable increase of the agency's budget since 2015 (Frontex, 2021; Fjørtoft, 2022). The migration crisis has become a theme for such border protection. It is mostly presented as a security issue because of ‘illegal migration’ that overstretched European asylum policy:

For Nehammer [Austrian Interior Minister], **efficient protection of external borders** that is in the interest of our common interior security and function Schengen cooperation is a priority. Moreover, the **Interior Minister demands a reform of the EU asylum system, in which the word asylum does not open the external border for illegal migration** and for which a more flexible notion of solidarity is emphasized. This is also supported by the four Visegrad countries, Poland, Czech Republic, Slovakia and Hungary, said Nehammer, who reaffirmed: “We explicitly reject an obligatory distribution of asylum seekers”. (AT, 2, 2020)

These quotes illustrate the Austrian government's approach to increased migration towards Europe and how it strategically places the demand for border protection and control in its public communication as a main solution. This shifts responsibility towards migrants and the EU, and therefore, out of reach. While such themes were first advanced by the FPÖ only, they have merged into the official government discourse up until now and so also point to the development of the ÖVP into an anti‐immigration party (Rheindorf and Wodak, 2018; Hadj Abdou and Ruedin, 2022). These patterns also suggest the normalization of a nationalist‐communitarian image that the government wants to portray. The Austrian government continually pits the idea of Europe under threat against the right to seek asylum, for example, demanding European solutions and evoking an exclusive European community.

This differs from the German case, where we find references to other crises as well, especially the COVID‐19 pandemic and climate crisis, and where we can also see references to more diverse border issues. Instead of a hyperfocus on border controls and border protection, governmental actors in Germany embed the migration crisis into the context of other ‘great challenges’. We find here that the German government highlights the ‘transboundary’ character of the last decade of European crises. In the coding scheme, this manifests as frequent overlapping between the migration crisis and COVID‐19, the economic crisis or climate crisis. The German government particularly uses phrases that refer to collaboration and multilateralism, presenting itself as an international collaborator. Foreign Minister Heiko Maas stated:

**All great challenges of the 21st century – pandemics, climate change, digitalization, migration – have something in common: They do not know borders, no national borders.** And this is why we need transboundary, that is, international solutions. This acknowledgement establishes **multilateralism as the cornerstone of our European foreign policy**. And, in turn, Europe needs to become more of a cornerstone in the multilateral system. (DE, 1, 2020)

Furthermore, German border communication highlights an area of tension between outer‐EU borders and inner‐EU space. In line with the German government's focus on the transboundary character of various defined European crisis events, we find statements of regret about inner‐EU border closures. Closing inner‐EU borders as a reaction to the pandemic is seen as an important, yet difficult situation evoked by others, if at all, as a statement by the German Foreign Minister illustrates:

**I do not see any neighbouring country which wants to be responsible for closing the borders.** 50‐kilometre traffic jams are also not in the interest of Poland – neither societally nor economically. (DE, 2, 2020).

The connection between crises and borders therefore suggests a more cosmopolitan outlook in the German government's communication. The focus on collaboration as well as crises as ‘transboundary’ (‘grenzüberschreitend’) is telling. This, however, does not necessarily mean a politics of open borders in Germany. While the officially intended image might be more moderate, in comparison to the Austrian government, German border communication, especially during the migration crisis, has also been considered volatile, as suggested in our quantitative analysis and by other research (Vollmer and Karakayali, 2018).

The second aspect that emerged as a dominant theme are cross‐border activities, which, as the analysis suggest, focus more strongly on neighbouring countries and so highlight a focus on ‘open borders’ within Europe. This theme has already been hinted at in our polarity analysis above. Cross‐border activities concern mostly times of stability where there is time to communicate, for example, about cultural and scientific exchanges, environmental and infrastructural issues, and trade relations, but also collaboration in terms of crime unrelated to migration and police work. Cross‐border activities are focused on the beneficial aspects of open borders within Europe. At the same time, they can be understood as part of inner‐European ‘debordering’ processes (see Schimmelfennig, 2021), but premised on strict external border protection. In the Austrian case, a typical example for cross‐border collaboration is police work, confirming the government's law and order approach to borders, here by the then Interior Minister Johanna Mikl‐Leitner who has been quoted in a press release about a cross‐border police initiative:

200 staff from Vienna, Lower Austria and Burgenland were engaged in this police operation. **They were supported by policewomen and policemen from Hungary and Slovakia** who employed mixed‐gender forces in the border area and increased pressure in their own country with focused actions. Five break‐in suspects, three after auto theft, two due to arrest warrants, and eight persons due to **illegal migration** were arrested during the police operation. (AT, 3, 2012)

Note also, that in 2012 (the year of the press release) the term ‘illegal migration’ is commonly used in Austrian press releases and remains so up until 2020, although the International Organization for Migration (IOM) has recommended using ‘irregular migration’ as a more neutral term (International Organization for Migration, 2019). The focus on police work, even cross‐border police work, highlights the Austrian government's approach to instrumentalize borders as state control (and that supposedly spun out of control during the migration crisis). Cross‐border police work is also an emerging theme when it comes to crime that is not related to migration, for example, in highly integrated areas.

Due to Upper Austria's geographical location in **the border triangle Austria, Germany and Czech Republic** the police **collaborate in the prevention and investigation of transboundary crime closely with the police organizations of its neighbours**. (AT, 4, 2013)

All in all, we find an Austrian ‘law and order’ approach to cross‐border activities as one characteristic in the government's communication.

In the case of the German government, our analysis suggests that border communication focuses on cross‐border collaboration in relation to crises as ‘global challenges’, ‘transboundary’ or ‘borderless’. This is also evident in more normative statements, where governmental actors inform about Germany's humanitarian engagement in conflict zones and in the demands that other countries join in these efforts. Often, the condition for this developmental support is beneficiary countries' efforts to secure their borders as a strategy against irregular migration.

**We are prepared to mobilize financial resources for developmental and humanitarian measures.** We have tasked our top national civil servants to coordinate closely with each other for the purposes of supporting the global efforts to limit the effects of the pandemic**. This also includes appropriate measures of border control management in accordance with regulations of single states** and, if necessary, support with the return of national citizens. (DE, 3, 2020).

Nevertheless, German and Austrian border communication share a focus on cross‐border collaboration in the issue of climate change/crisis, resources and cultural exchanges, aspects that are considered more long‐term ‘governing as usual’. Investment and development of water infrastructure play a role for regional development and infrastructure, for example, as the case of Austria illustrates:

The expansion of **inter‐state traffic connections** took centre stage in the visit of the Czech traffic minister, Antonin Prachar, to his Austrian colleague, Doris Bures. The [Austrian] traffic minister mentioned the **very positive work exchange**. (AT, 5, 2014)

More generally, in the case of Austria, communication about cross‐border collaboration, such as cultural exchanges, financial and infrastructural investment, are highlighted as conducive to economic growth in the region, creating a mutually beneficial situation for Austria and its neighbouring countries. In the German case this focus is further expressed in relation to infrastructure investment as a form of international economic and social development:

**Transboundary water management is a vital component of developmental policy and contributes to reducing poverty.** By agriculturally using water resources together, we can contribute to water, energy and food safety. (DE, 4, 2013).

In this sense, both German and Austrian governmental border communication emphasize the benefits of cross‐border activities, enabled via regulated permeable borders, when it comes to economic growth, development or trying to mitigate causes of flight, such as poverty. This also implies that openness is regulated and ‘approved’ by the government where it fits governmental interests. Furthermore, expressions of openness through cross‐border collaboration for economic growth are consistently separated from border communication in relation to migration.

## V Discussion: Border Communication as a Strategy

The ways in which both governments communicate about borders can be explained through, first, the specific national contexts, which contextualize the differences between both cases: Germany through seemingly collaborative, multilateral efforts; Austria with a focus on security or law and order. These differences can likely be attributed to the varying degrees in which right‐wing party politics has been able to influence government communication: while Germany was governed by a dominant centre‐right party with varying coalition partners, anti‐immigration parties were more dominant in the Austrian government, with the ÖVP turning in this direction and the FPÖ as a right‐wing party (Hadj Abdou and Ruedin, 2022).

Yet, beyond these differences, our findings show how governments instrumentalize borders and communicate their specific degrees of openness and closedness across different policy fields. Our analysis highlights that both governments selectively apply communication of politics of closed and open borders. Both governments, but especially the case of Austria, highlight the urgency to protect external EU borders. This might be understood as a strategic attempt to point to the EU and other EU Member States with responsibilities of border protection. This focus furthermore implies that a ‘borderless’ Europe is not possible until it is closed to the outside, as can also be seen in the focus on national borders despite EU freedom of movement. Therefore, governmental border communication does not follow along the lines of cosmopolitan and communitarian cleavages (see Strijbis, et al., 2020) or a clear debordering within the EU, while external rebordering is demanded (see Schimmelfennig, 2021). The strategical application of notions of open and closed borders therefore makes the practice of filtering and selection by governments visible on a broader scale (see Johnson et al., 2011, p. 66).

Furthermore, our analysis raises interesting questions regarding the construction of ‘open borders’. Openness is communicated in a way that suggests control through specific collaborations of different institutions or trade. Instead, governments' focus on national borders is prevalent, while with increasing European integration, a focus on external borders is obvious. Openness is, then, communicated as a controlled process for specific purposes and mainly within the EU, or from the EU towards the rest of the world, for example, through humanitarian support. In other words, on the basis of our data it is hard to understand openness as translating into a ‘borderless’ Europe.

## Conclusion

We have explored governmental border communication in Austria and Germany during a decade of crises. Based on our approach, drawing from more constructivist border studies, our analysis strongly suggests that via communication, governments strategically apply varying degrees of border permeability that are conflicting, cosmopolitan and communitarian, as well as re‐ and debordering at the same time. Within approaches to European dis‐/integration, governments often ‘disappear’ behind the broader focus on the state apparatus. The competencies of governments might be more short‐term within the bigger picture of European dis‐/integration. Yet, a better understanding of how ‘applied’ border politics is communicated has enabled us to draw a more fine‐grained and nuanced picture of border politics and provides contextual understanding that might even help us to better understand the dynamics of European dis‐/integration as reactive, not always intentional, processes. To better understand these aspects, further research should include more cases, be designed to manage multilingual samples and test causal relationships. Furthermore, our analysis cannot address the scope through which border communication is adapted to potential, diverse target audiences. More research is urgently needed to gain deeper insights into this relationship.

Notwithstanding these limitations, our analysis finally points to a rather disconcerting governmental understanding of ‘openness’ in Europe. Within the last decade the notion of a ‘borderless’ European discourse might have shifted towards an ‘open and controlled’ Europe. The recent crises might have heightened a sense of socio‐political instability and perceived threat which is also expressed in governmental border communication. As governments respond to these circumstances, the notion of Europe as a controlled space might be on its way to becoming a ‘new normal’.

## Supporting information


**Appendix A1.** **Model Terms for Border Topic in the Austrian Governments' Press Releases**
Appendix A2. Model Terms for Border Topic in the German Governments' Press Releases
Appendix A3. Codes Emerging from Qualitative Analysis
Appendix A4. Translation and Sources for Quoted Press Release Material
Appendix A5. Documentation of How We Arrived at the Threshold for Model Terms

## Footnotes

^1^
For details on how we arrived at the specific threshold of 10, please see Appendix A5.

^2^
Please see Appendix A3 for an overview of the different codes.

^3^
Quotes translated from German by the authors. For reasons of legibility and space, we include the original German versions of the quoted material in the Appendix (see Appendix A4).
