# Supplementary material for: A Thin Line: Governmental Border Communication in Times of European Crises
Source: J Common Mark Stud. 2022 Aug 15;61(3):597–615. doi: 10.1111/jcms.13398 (PMC10947545; doi:10.1111/jcms.13398)
Supplement: Supplementary file 1 — Appendix A1. Model Terms for Border Topic in the Austrian Governments' Press Releases Appendix A2. Model Terms for Border Topic in the German Governments' Press Releases Appendix A3. Codes Emerging from Qualitative Analysis Appendix A4. Translation and Sources for Quoted Press Release Material Appendix A5. Documentation of How We Arrived at the Threshold for Model Terms [file JCMS-61-597-s001.pdf]

Appendix Material A1: Model Terms for Border Topic in the Austrian Governments' Press Releases

German Original	English Translation
hinweg, spielfeld, nickelsdorf, gigalinern, assistenzeinsatz, gesundheitsbehördlichen, nacheile, sicherheitspolizeilichen, küstenwache, kriminalität, zentralasiatischen, stoflen, überschreiten, überschritten, drohnen, halt, weit, assistenzeinsatzes, kontrollen, fremdenpolizei, kontrollieren, zusammenarbeit, ungarn, kennt, östlichen, eisernen, frontex, vorhangs, hinaus, zentralasien, polizei, slowenien, migration, zulassung, einreise, schlepperei, brenner, entlang, bekämpfung, polizisten, illegaler, überwinden, polizeiliche, burgenländischen, soldaten, wegfall, gesundheitsbehörden, verstärkung, überwachung, illegale, illegalen, ministerkonferenz, eingesetzt, katastrophenhilfe, gemeinsame, nahe, erleichtert, polizistinnen, einsatz, behörden, luftraum, tschechien, ungarischen, tätigkeiten, slowakei, nationalen, sicherheit, innenminister, österreichs, sicherung, kontrolliert, soldatinnen, kräfte, donau, terrorismus, organisierte, kooperation, sowie, verstärkte, grünen, italien, unterstützung, sprechen, fahrzeuge, aufgaben, klare, kilometer, bundesheer, möglichen, burgenland, durchgeführt, herausforderungen, bekämpfen, verteidigungsministerin, liechtenstein, gemeinsamen, bereich, kontrolle, kroatien, beim, schutz, bundesheeres, immer, griechenland, schweiz, offene, möglichkeiten, übung, lage, unseren, mobilität, kooperationen, unserer, flüchtlinge, schützen, tiroler, deutschland, raum, staaten, nationale, eu, innenministerin, kulturellen, austausch, europäischen, kampf, gewährleistet, unseres, vernetzung, landes, unterstützen, regionale, sagte, europa, unsere, täglich, amtskollegen, ermöglicht, rahmen, bevölkerung, beispielsweise, europas, verordnung, beiden, notwendig, sei, maflnahmen, verstärkt, sichern, region, verhindern, sorgen, innerhalb, ab, bekannt, deutschen, österreichischen, zuge, anfang, geschaffen, dialog, transparenz, situation, verteidigungsminister, auswirkungen, personen, ebene, arbeiten, gemeinsam	across, spielfeld, nickelsdorf, gigaliners, assistance deployment, health, hot pursuit, security police, coast guard, crime, central asian, stoflen, cross, exceeded, drones, stop, far, assistance, controls, alien, police, control, cooperation, hungary, knows, eastern, iron, frontex, curtain, out, central asia, police, slovenia, migration, admission, entry, smuggling, burner, along, combat, police, illegal, overcome, police, burgenland, soldiers, omission, health, authorities, reinforcement, surveillance, illegal, ministerial, deployed, disaster, assistance, joint, close, facilitated, policemen, deployment, authorities, airspace, czech, hungary, activities, slovakia, national, security, interior, austria, security, controlled, female, soldiers, forces, danube, terrorism, organized, cooperation, as well as, reinforced, green, italy, support, speak, vehicles, tasks, clear, kilometers, federal, army, possible, burgenland, carried, challenges, combat, defense, minister, liechtenstein, joint, area, control, croatia, in, protection, federal, army, always, greece, switzerland, open, opportunities, exercise, situation, our, mobility, cooperation, our, refugees, protect, tyrol, germany, space, states, national, eu, interior, minister, cultural, exchange, europe, fight, ensured, our, networking, country, support, regional, said, europe, our, daily, officemates, enabled, framework, population, for, example, europe's, regulation, both, necessary, be, measures, strengthened, secure, region, prevent, care, within, starting, known, german, austrian, added, beginning, created, dialogue, transparency, situation, defense, minister, impact, people, level, work, joint

Appendix Material A2: Model Terms for Border Topic in the German Governments' Press Releases

**German Original**	**English Translation**
hinweg, reporter, überschreitenden, überschreitende, halt, türkisch-syrischen, deutsch-österreichischen, innerdeutschen, schengener, öncüpinar, überschreitendes, hinausstrahlen, rechtsverstöße, deutsch-polnische, zollbehörden, kennt, innerdeutsche, zwischengesellschaftliche, kriminalität, überschreiten, flüchtlingslager, zusammenarbeit, deutsch-polnischen, überschritten, polizei-, lebensmittelüberwachung, dadaab, amtlichen, vorübergehenden, modellcharakter, strengeren, bundespolizei, deutsch-tschechischen, koordinatorin, überwinden, bekämpfung, nahe, stoflen, öffnung, veröffentlichen, entlang, kontrollen, asylerstanträge, polen, ehemaligen, strenge, tschechien, entfernt, bundesverfassungsgericht, dänemark, weit, atomkraftwerke, kulturelle, tschechischen, hinaus, österreich, deutsch-französischen, gaza, band, kontrolle, planeten, kennen, jenseits, schweiz, engen, versorgungssicherheit, migration, klare, ärzte, nationale, innerhalb, virus, frankreich, gesetzt, behörden, kilometer, betreffen, festgestellt, europa, städtebaus, schweinepest, berufsbildung, asp, früheren, eingehalten, effektive, illegalen, pressefreiheit, regionale, kreativität, nähe, terrorismus, europäischen, kooperation, beiden, syrischen, bundesinnenminister, einhaltung, reisen, festgelegt, möglichkeiten, zusammenarbeiten, libanon, syrien, bestimmte, gemeinsamen, verkehr, insbesondere, nachbarn, verbindet, besseren, geltenden, denen, polizei, gelten, sicherheit, darf, auswirkungen, gemeinsame, israel, republik, waffen, europäische, schutz, verbinden, vertrag, bestehenden, thüringen, seiten, bekämpfen, kooperationen, austausch, nationalen, raum, genannten, europas, verpflichtet, italien, macht, mobilität, beider, regeln, vorschriften, hilfe, urteil, eng, chancen, unseres, grundlagen, liegen, gebieten, etwa, wirksam, beitragen, eu, eu-kommission, lösungen, bereich, ukraine, pandemie, erforderlich, humanitäre, tage, zugleich, abkommen, beispiel, immer, türkei, situation, bürger, deutschlands, maiziËre, vorgehen, eigenen, geschaffen, freiheit, gilt, arbeiten, zeiten, einrichtung, bayern, möglich, menschen, flüchtlinge, besonders, bundesländer, ermöglicht	across, reporter, crossing, crossing, stop, turkish-syrian, german-austrian, inner-german, schengen, öncüpinar, crossing, beaming out, violations, german-polish, customs, knows, inner-german, inter-societal, crime, transgression, refugee camp, cooperation, german-polish, transgression, police, food surveillance, dadaab, official, temporary, model, stricter, federal police, german-czech, coordinator, overcome, combat, close, stoflen, opening, publish, along, controls, asylum, applications, poland, former, strict, czech, removed, federal constitutional court, denmark, far, nuclear, cultural, czech, beyond, austria, german-french, gaza, tape, control, planet, know, beyond, switzerland, narrow, supply, migration, clear, doctors, national, within, virus, france, set, authorities, kilometer, concern, noted, europe, urban, swine, plague, vocational, asp, previous, complied, effective, illegal, press, freedom, regional, creativity, proximity, terrorism, european, cooperation, both, syrian, federal, interior, compliance, travel, set, opportunities, cooperate, lebanon, syria, certain, joint, traffic, particular, neighbor, connects, better, applicable, those, police, apply, security, may, impact, joint, israel, republic, weapons, european, protection, connect, treaty, existing, thuringia, sides, combat, cooperation, exchange, national, space, named, europe, committed, italy, power, mobility, both, rules, regulations, aid, judgment, narrow, opportunities, our, bases, lie, areas, about, effective, contribute, eu, eu commission, solutions, area, ukraine, pandemic, required, humanitarian, days, at, same, agreement, example, ever, turkey, situation, citizen, germany, maiziËre, proceed, own, created, freedom, applies, work, times, facility, bavaria, possible, people, refugees, especially, federal, states, enables

Appendix Material A3: Codes emerging from qualitative analysis

As it is common practice in the inductive coding process, the below categories are not mutually exclusive but can overlap. A3 therefore dominantly serves as a transparent overview over the coding process

**Coding categories**		**DE**	**AT**	**Total**
**Cross-border activities**				
	Cross-border exchange	2,3%	3,7%	3,1%
	Cross-border developmental/humanitarian/other support	1,2%	0,5%	0,8%
	Cross-border crime (non-migration)	1,8%	5,3%	3,6%
	Cross-border standards/regulations	1,2%	0,5%	0,8%
	Cross-border trade	1,8%	0,0%	0,8%
**Bordering**				
	border protection	0,0%	2,1%	1,1%
	irregular migration, trafficking, smuggling	0,0%	3,7%	1,9%
	Asylum issues	1,2%	0,5%	0,8%
	Border controls	0,6%	7,4%	4,2%
**Types of borders**				
	external EU borders	1,2%	3,7%	2,5%
	Inner-EU borders	1,8%	2,1%	1,9%
	sea/air borders	0,6%	0,0%	0,3%
	national borders	7,6%	13,3%	10,6%
**Types of crises**				
	food crises	1,8%	0,0%	0,8%
	Russia/Ukraine crisis	0,6%	0,5%	0,6%
	non-COVID health crisis/epidemic	8,2%	0,0%	3,9%
	Historical crisis	1,2%	2,1%	1,7%
	Climate and nuclear crisis and resources	10,5%	3,2%	6,7%
	General crisis or crisis as conflict or developmental	7,6%	1,1%	4,2%
	Economic crisis	9,9%	11,2%	10,6%
	Humanitarian crisis	3,5%	1,6%	2,5%
	Transboundary crisis	11,7%	2,7%	7,0%
	COVID-19	8,2%	4,8%	6,4%
	Migration/refugee crisis	10,5%	23,4%	17,3%
	Terrorism	5,3%	6,4%	5,8%
**Total**		**100%**	**100%**	**100%**

Appendix Material A4: Translation and sources for quoted press release material

AT, 1, 2016
[...] ‘Europa ist gefordert das Sterben im Mittelmeer zu beenden. Wir müssen die momentane Verschnaufpause durch die Schließung der Westbalkan-Route und das Abkommen mit der Türkei nutzen um eine europäische Lösung zu finden. Dafür werde ich mich auch beim heutigen EU-Außenministerrat in Luxemburg erneut einsetzen. **Wir dürfen die Entscheidung wer es nach Europa schafft nicht mehr kriminellen Schlepperbanden überlassen**. Die Rettung aus dem Mittelmeer darf auch nicht mehr verbunden sein mit einem Ticket nach Mitteleuropa. **Daher müssen wir illegale Migration und kriminelle Schlepperbanden entschieden bekämpfen und die EU-Außengrenzen gemeinsam ordentlich sichern.** Zugleich sollten wir viel mehr Hilfe vor Ort leisten legale Fluchtwege über Resettlement-Programme eröffnen und mehr Druck auf Herkunftsländer ausüben.[...]’
[Austrian Minister for Europe and External Affairs, Sebastian Kurz, 2016, https://www.ots.at/presseaussendung/OTS_20160620_OTS0010/bundesminister-kurz-europa-ist-gefordert-das-sterben-im-mittelmeer-zu-beenden]

AT, 2, 2016
[...]Vorrangig ist für Nehammer ein **effizienter Außengrenzschutz**, der im Interesse unserer gemeinsamen inneren Sicherheit und einer funktionierenden Schengenkooperation steht. Darüber hinaus fordert **der Innenminister eine EU-Asylreform, bei der das Wort Asyl nicht die Außengrenze für illegale Migration öffnen dürfe** und bei der eine flexible Solidarität im Zentrum stehen solle. Dies wird auch unter anderem von den vier Visegrad-Staaten Polen, Tschechien, Slowakei und Ungarn unterstützt, sagte Nehammer, der bekräftigte: ‘Eine verpflichtende Verteilung von Asylwerbern lehnen wir klar ab.’ [...]
[Austrian Interior Minister, 2020, https://www.ots.at/presseaussendung/OTS_20200212_OTS0193/nehammer-schutz-der-aussengrenzen-und-frontex-forcieren-ist-das-gebot-der-stunde]

DE, 1, 2020
‘[...]**Alle großen Herausforderungen des 21. Jahrhunderts – Pandemien, Klimawandel, Digitalisierung, Migration – haben eines gemeinsam: Sie kennen keine Grenzen, keine nationalen Grenzen.** Und deshalb brauchen wir dafür grenzüberschreitende, eben internationale Lösungen. Es ist diese Einsicht, die den **Multilateralismus zum Grundpfeiler unserer europäischer Außenpolitik** macht. Und umgekehrt muss Europa selbst noch stärker zum Grundpfeiler des multilateralen Systems werden.[...]’
[German Foreign Minister, Heiko Maas, 2020, https://www.auswaertiges-amt.de/de/newsroom/maas-boko-paris/2379972]

DE, 2, 2020
‘[...]Ich sehe kein Nachbarland, das leichtfertig dazu beitragen will, dass wir die Grenzen dicht machen. 50-Kilometer-Staus sind ja auch nicht im Interesse Polens – weder gesellschaftlich noch wirtschaftlich.[...]’ [German Foreign Minister, Heiko Maas, 2020, https://www.auswaertiges-amt.de/de/newsroom/maas-tagesspiegel/2411294]

AT, 3, 2012
[...]Bei diesem Einsatz waren rund 200 Polizistinnen und Polizisten in Wien in Niederösterreich und im Burgenland im Einsatz. **Sie wurden von Polizistinnen und Polizisten aus Ungarn und der Slowakei unterstützt, die im grenznahen Bereich gemischte Streifen einsetzten und in Schwerpunktaktionen im eigenen Land den Kontrolldruck erhöhten.** Bei dem Einsatz wurden fünf Verdächtige nach Einbrüchen drei nach Kfz-Diebstahl zwei aufgrund eines Haftbefehls und acht Personen wegen **illegaler Migration** vorläufig festgenommen [...].
[Austrian Police Spokesperson Michael Takács, 2012, https://www.ots.at/presseaussendung/OTS_20121116_OTS0246/erfolgreicher-soko-ost-schwerpunkteinsatz-18-festnahmen-in-einer-nacht]

AT, 4, 2013
‘[...] Aufgrund Oberösterreichs geographischer Lage **im Drei-Länder-Eck Österreich**, Deutschland und Tschechien arbeitet die Polizei in der Verhinderung und **Aufklärung von grenzüberschreitenden Straftaten eng mit den Polizeiorganisationen der Nachbarländer zusammen.** [...].
[General Director for Public Safety, Konrad Kogler, 2013, https://www.ots.at/presseaussendung/OTS_20130424_OTS0268/generaldirektor-fuer-die-oeffentliche-sicherheit-professionelle-und-erfahrene-polizei]

DE, 3, 2020

[...]Wir sind bereit, Finanzmittel für entwicklungspolitische und humanitäre Maßnahmen zu mobilisieren. Wir beauftragen unsere Spitzenbeamten, sich zur Unterstützung der **globalen Anstrengungen**, die Auswirkungen der Pandemie zu begrenzen, eng abzustimmen. **Dazu gehören angemessene Maßnahmen des Grenzmanagements in Übereinstimmung mit einzelstaatlichen Regelungen** und erforderlichenfalls Hilfeleistung bei der Rückführung von Staatsangehörigen [...].

[German Federal Chancellor Office, 2020, https://www.bundeskanzlerin.de/bkin-de/aktuelles/ausserordentliches-gipfeltreffen-der-staats-und-regierungschefs-der-g20-erklaerung-zu-covid-19-1735452]

AT, 5, 2018

'[...]Der Ausbau der **zwischenstaatlichen Verkehrsverbindungen** stand im Zentrum des Arbeitsbesuchs des tschechischen Verkehrsministers Antonin Prachar bei der seiner österreichischen Amtskollegin Doris Bures. Die Verkehrsministerin sprach im Anschluss von einem sehr positiven Austausch über den jeweiligen Stand der Arbeiten: Die neue tschechische Regierung räumt dem Verkehrsbereich Priorität in den bilateralen Beziehungen zu Österreich ein.

[Austrian Minister for Traffic, Innovation, and Technology, Doris Bures, 2018, https://www.ots.at/presseaussendung/OTS_20180620_OTS0171/danube-day-2018-aktionstag-fuer-eine-saubere-donau]

DE, 4, 2013

[...]Grenzüberschreitendes Wassermanagement ist integraler Bestandteil der Entwicklungspolitik und trägt zur Armutsminderung bei. Indem Wasserressourcen gemeinsam bewirtschaftet werden kann ein wichtiger Beitrag zur Wasser- Energie und Ernährungssicherung geleistet werden.[...]

[German Ministry for Development, 2013. *The ministry's archive does not reach back to this time. The press release can be accessed upon request.*]

Appendix Material A5: Documentation of how we arrived at the threshold for model terms

In order to filter out such press releases that do not represent the border topic substantively, we proceeded in steps, looking into different threshold values for the number of model terms (= terms defining the border theme; see Appendix Material A1 and A2) to understand its influence on the results. As the histogram shows, the highest density of model terms is found below 50; most press releases, thus, mention model terms (n_cris) up to 50 times in the text.

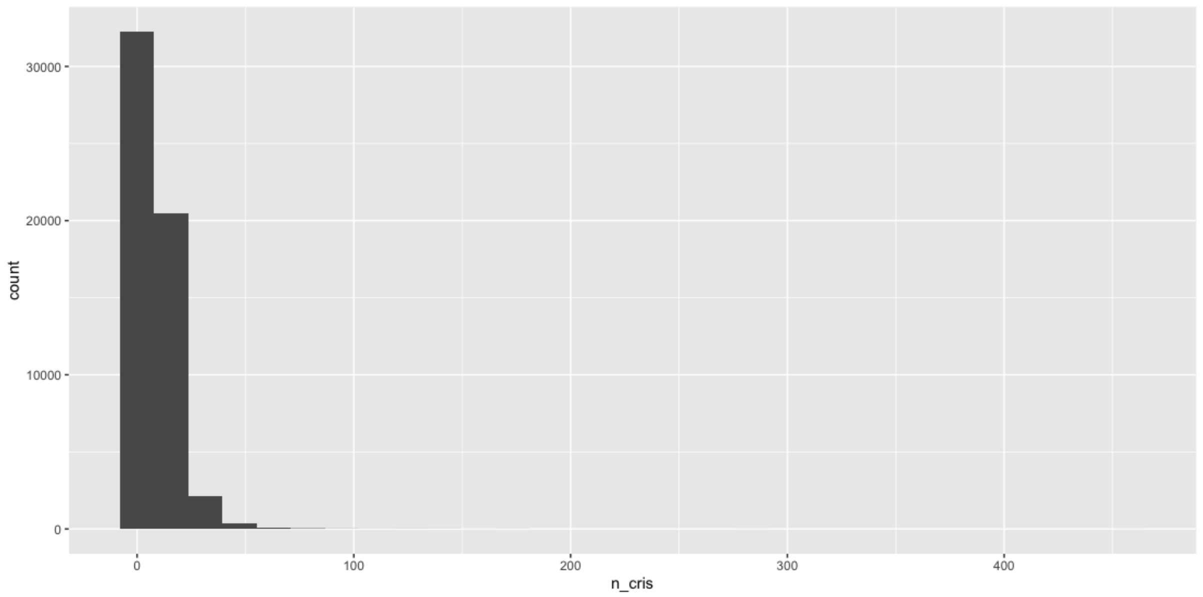

In the following, we started from 1 (> 0), proceeding in steps of 5; thus first excluding only press releases that did not mention model terms at all; then excluding such press releases mentioning only up to 4 of our model terms in the text, etc. Density of model terms is the number of model terms mentioned in a press release. As a result, we decided to use a density of 10 model terms for our analysis as this solution already shows the trends in polarity scalings emerging later while still representing a clear and well-interpretable solution. LSX does produce a lot of random noise (see Watanabe, 2021); given that our results are validated by the qualitative in-depth coding, we regard this a valid procedure.

a) Permeability and state of affairs re: borders in Austrian press releases

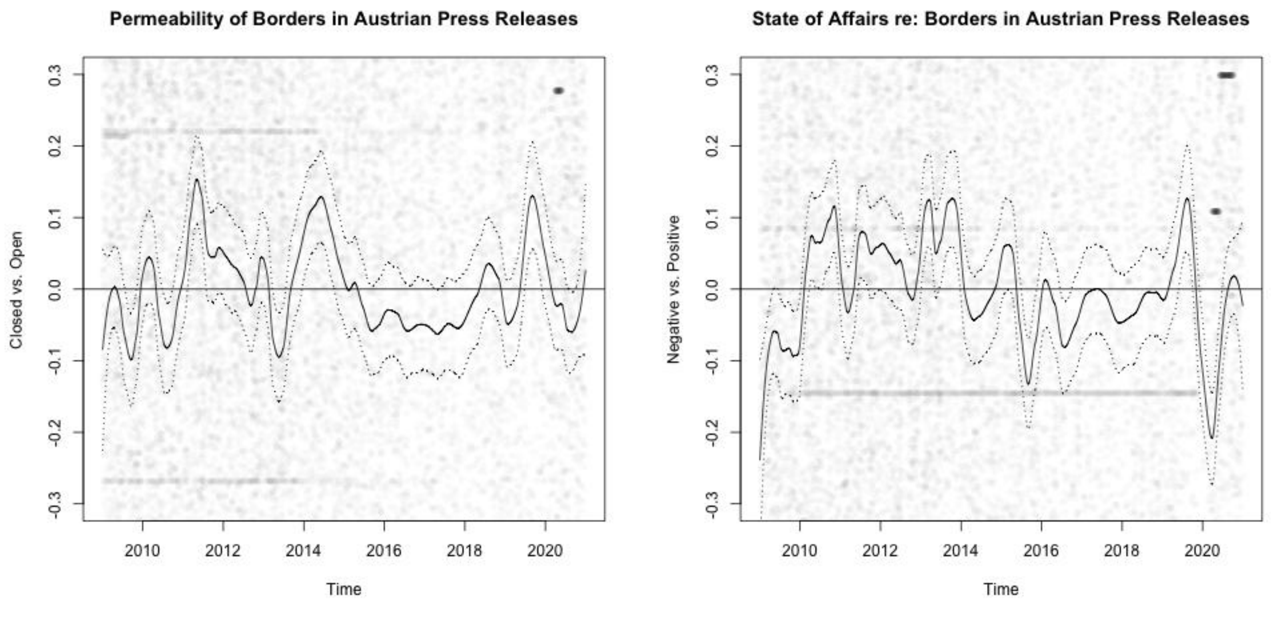

Note: Density of model terms > 0; n = 26634

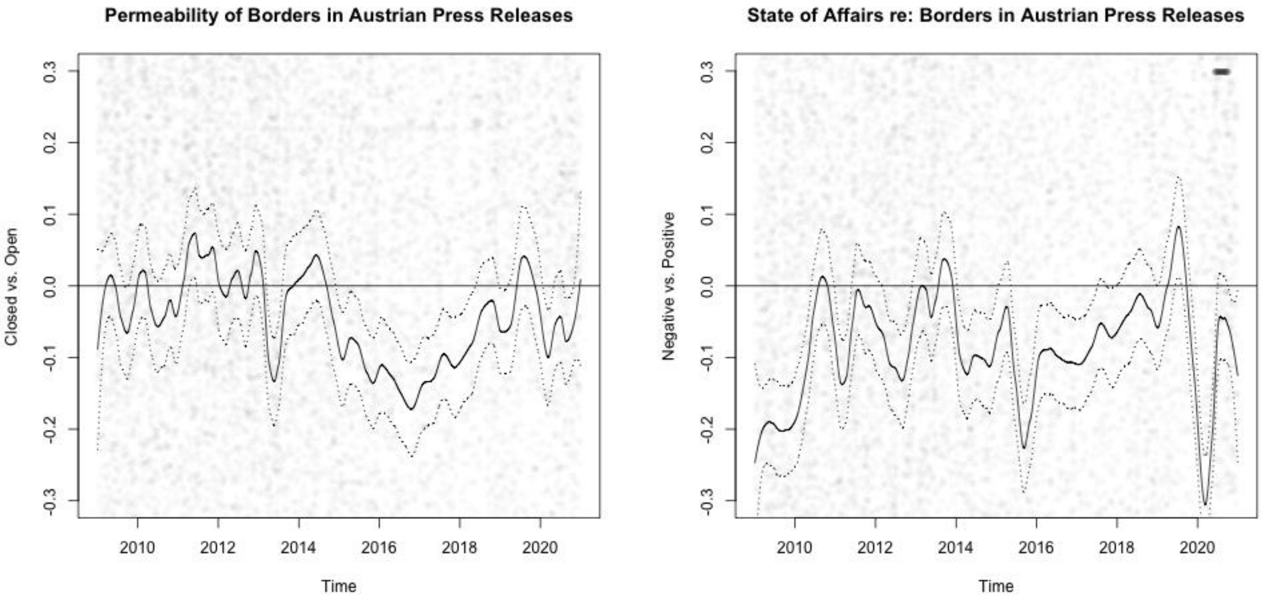

Note: Density of model terms > 4; n = 17992

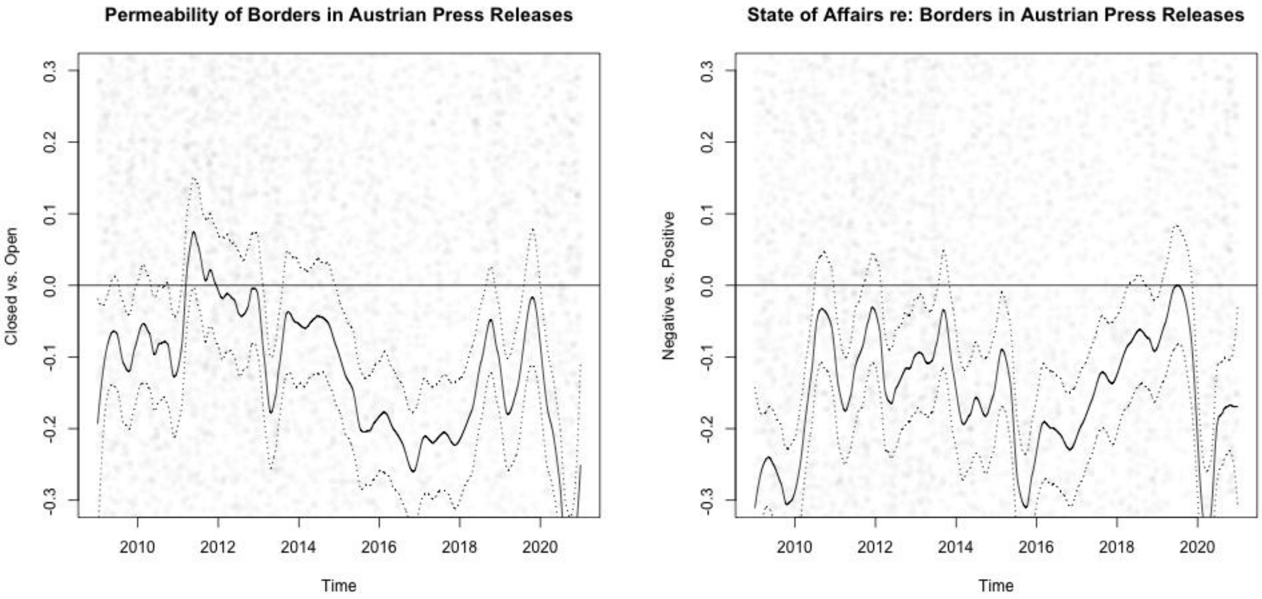

Note: Density of model terms > 9, 9949

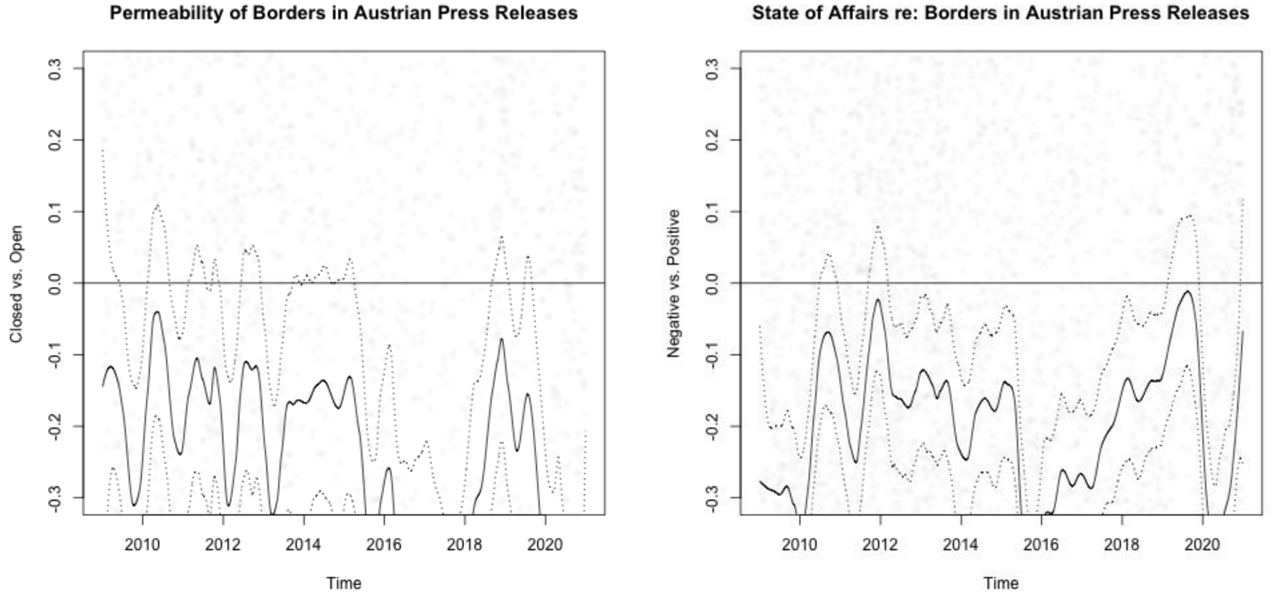

Note: Density of model terms > 14; n = 5312

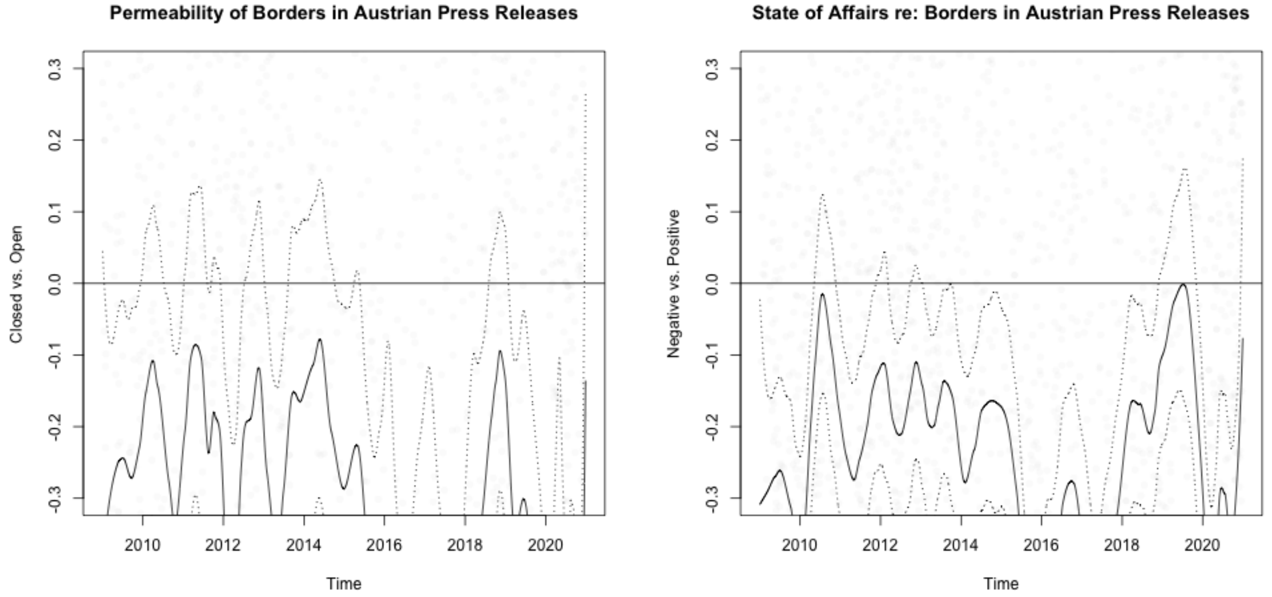

Note: Density of model terms > 19; n = 2708

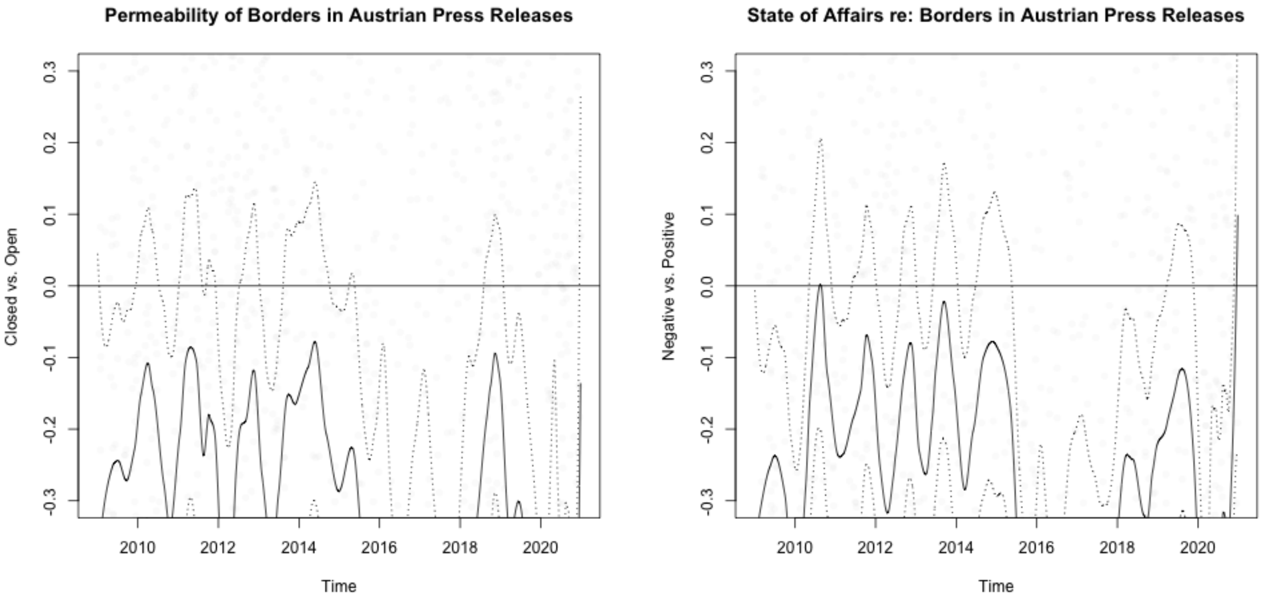

Note: Density of model terms > 24; n = 1389

b) Permeability and state of affairs re: German press releases with increasing thresholds for model terms density

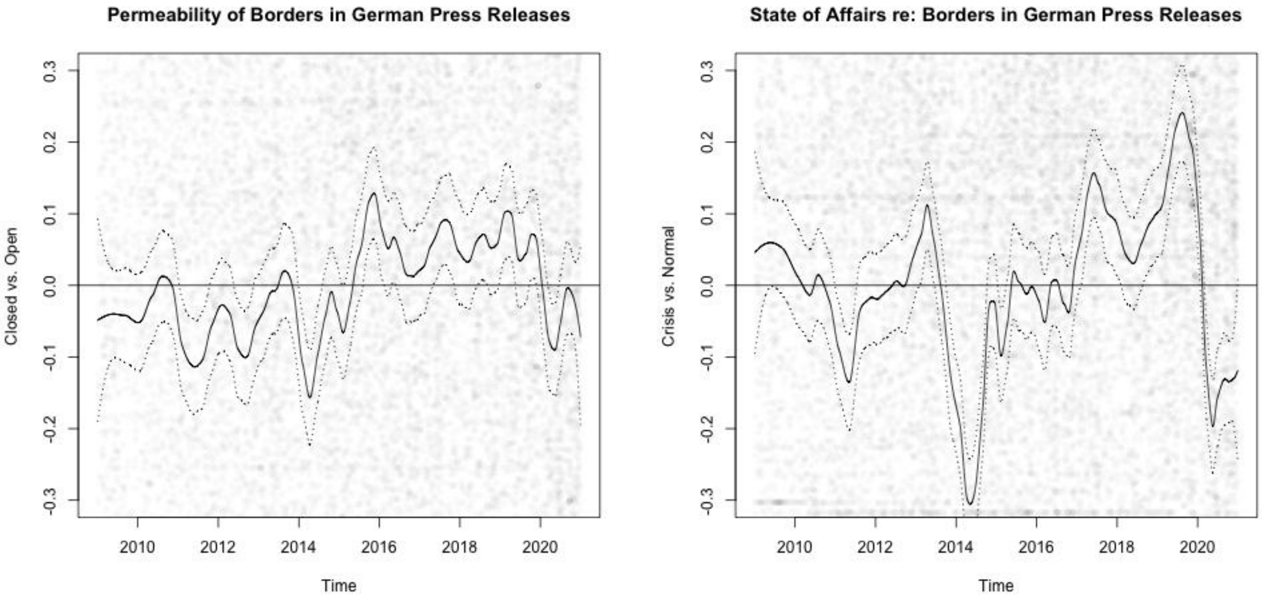

Note: Density of model terms > 0; n = 26034

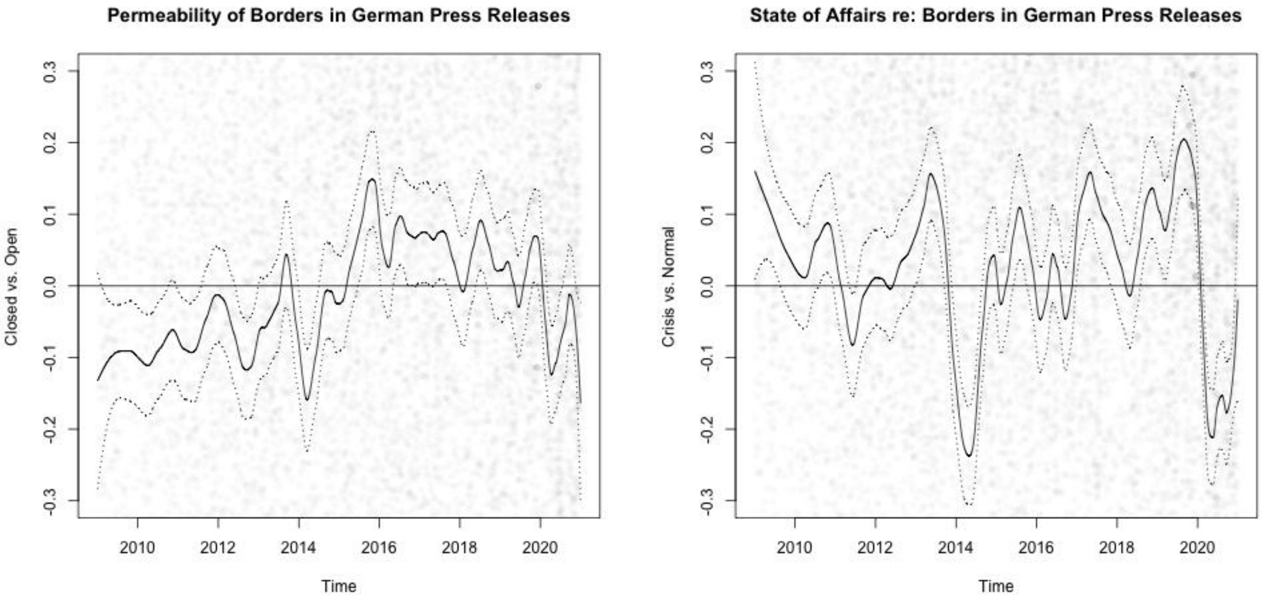

Note: Density of model terms > 4; n = 14758

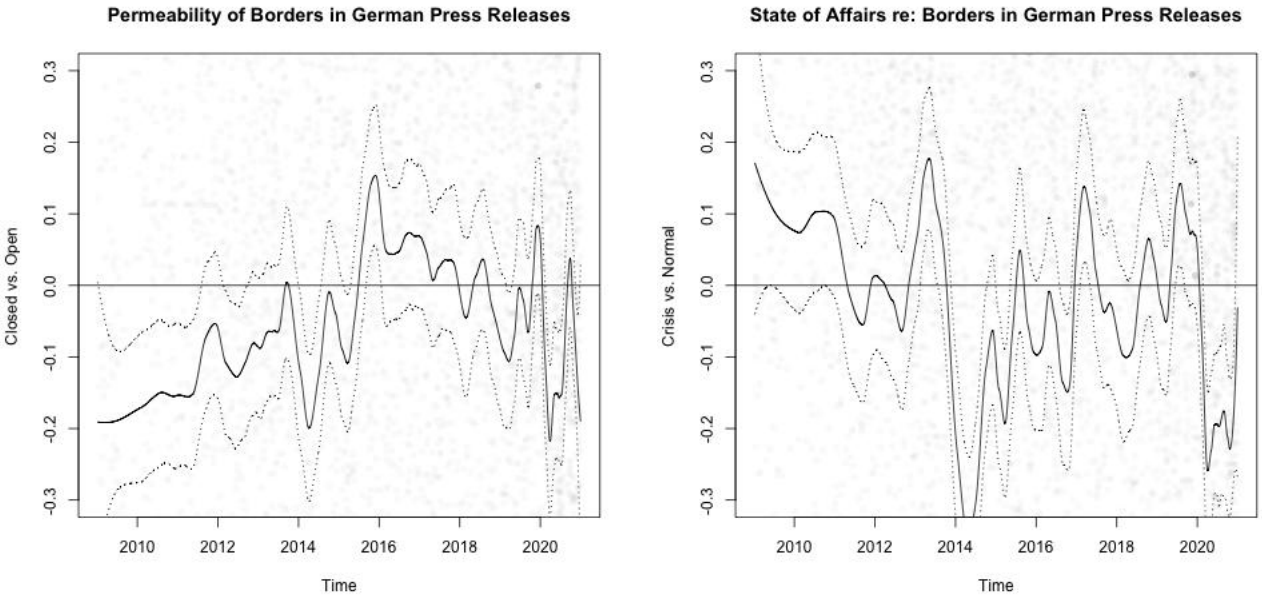

Note: Density of model terms > 9; n = 5844

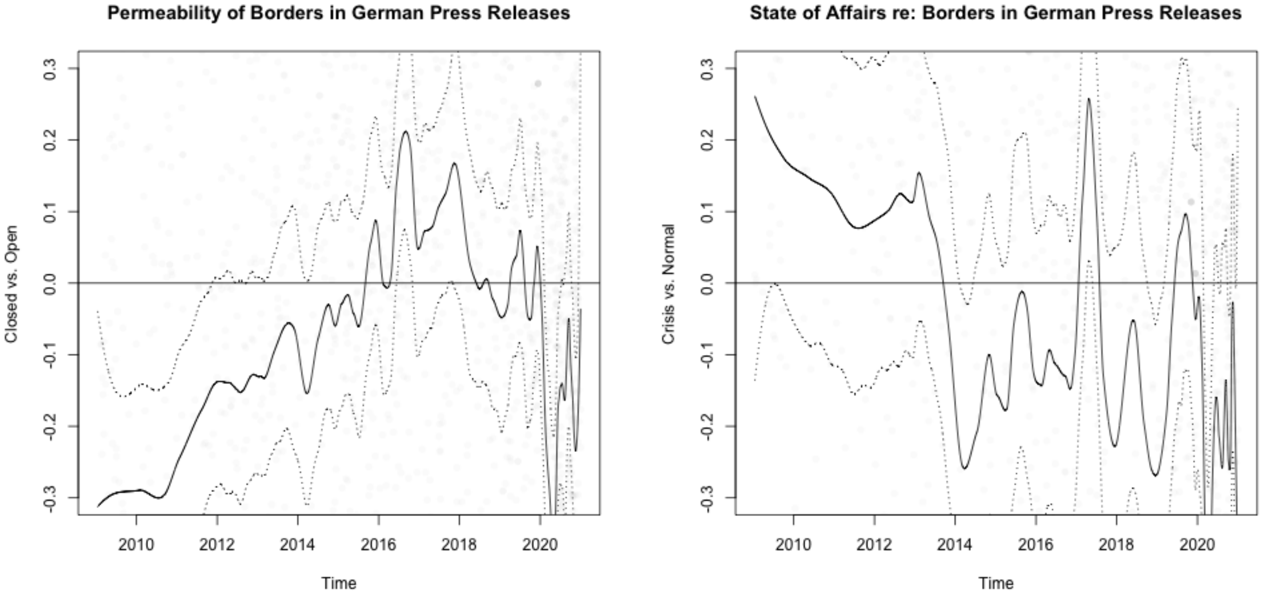

Note: Density of model terms > 14; n = 2293

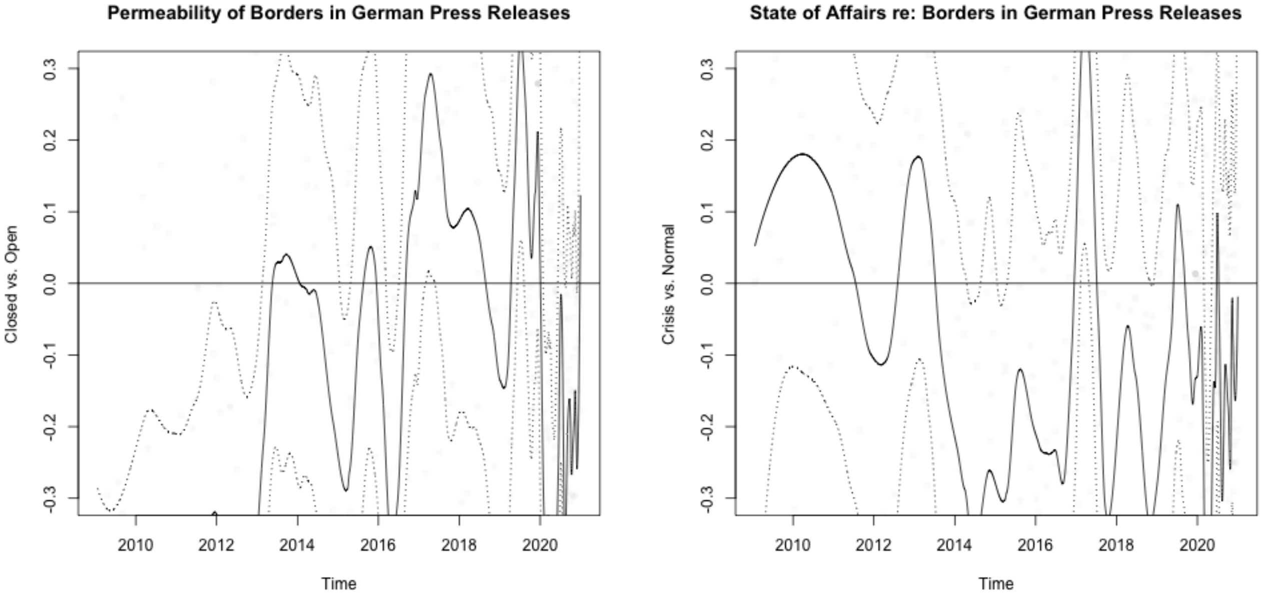

Note: Density of model terms > 19; n = 1032

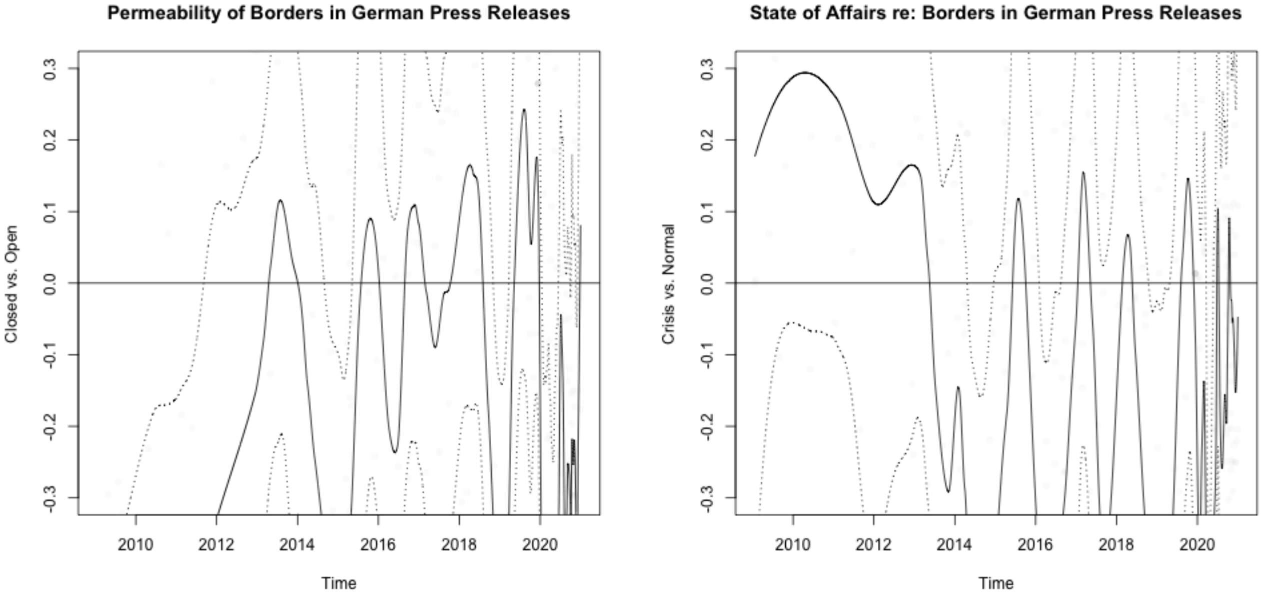

Note: Density of model terms > 24; n = 540
